# Barriers to HIV remission research in low- and middle-income countries

**DOI:** 10.7448/IAS.20.1.21521

**Published:** 2017-06-05

**Authors:** Theresa Rossouw, Joseph D. Tucker, Gert U. van Zyl, Kenly Sikwesi, Catherine Godfrey

**Affiliations:** ^a^ Institute for Cellular and Molecular Medicine, Department of Immunology, University of Pretoria South Africa, Pretoria, Republic of South Africa; ^b^ Institute for Global Health and Infectious Diseases, UNC Chapel Hill, Chapel Hill, NC, USA; ^c^ UNC Project-China, Institute for Global Health and Infectious Diseases, Sikwese African Community Advisory Board (AFROCAB), Guangzhou, China; ^d^ Division of Medical Virology, Stellenbosch University and NHLS Tygerberg, Cape Town, South Africa; ^e^ National Health Laboratory Services, Tygerberg, Cape Town, South Africa; ^f^ Coordinator of the African Community Advisory Board (AFROCAB), Lusaka, Zambia; ^g^ National Institute of Allergy and Infectious Diseases, National Institutes of Health, Bethesda, MD, USA

**Keywords:** HIV eradication, HIV cure, HIV remission, resource limited, low- and middle-income countries, immune system

## Abstract

**Introduction**: HIV eradication and remission research has largely taken place in high-income countries. In low- and middle-income countries (LMIC), there may be factors that have a substantial impact on the size of the latent HIV reservoir and the immunological response to infection. If a curative strategy is to be available to all HIV-infected individuals, these factors must be understood.

**Methods**: We use a scoping review to examine the literature on biological factors that may have an impact on HIV persistence in LMIC. Three databases were searched without date restrictions.

**Results**: Uncontrolled viral replication and higher coinfection prevalence may alter the immunological milieu of individuals in LMIC and increase the size of the HIV reservoir. Differences in HIV subtype could also influence the measurement and size of the HIV reservoir. Immune activation may differ due to late presentation to care, presence of chronic infections, increased gut translocation of bacterial products and poor nutrition.

**Conclusions**: Research on HIV remission is urgently needed in LMIC. Research into chronic immune activation in resource poor environments, the immune response to infection, the mechanisms of HIV persistence and latency in different viral clades and the effect of the microbiological milieu must be performed. Geographic differences, which may be substantial and may delay access to curative strategies, should be identified.

## Introduction

Access to antiretroviral therapy (ART) has improved the lives of millions affected by HIV, but HIV remains incurable. In 2015, there were an estimated 36 million people living with HIV (PLHIV), most of whom live in low- and middle-income countries (LMIC). Worldwide, approximately half of HIV-infected individuals accessed HIV treatment but there are wide differences between countries in the number of HIV-infected individuals on treatment [1]. Recent data have spurred the World Health Organization (WHO) to recommend ART for all HIV-infected individuals [2]; thus, there will be more individuals receiving treatment in the near to medium future. HIV treatment is lifelong and strict adherence to treatment regimens is required for successful therapy. The burden of care for treatment programmes in countries with limited resources is enormous and will continue to grow. Moreover, the immunological damage due to HIV infection and ongoing viral replication leads to a cascade of events that are associated with important noninfectious morbidities which will likely stress healthcare resources even further. Sustained remission of HIV without the need for ART, if attainable, will be critical to the overall health of the population and will allow for a broader distribution of resources.

**Figure 1.**
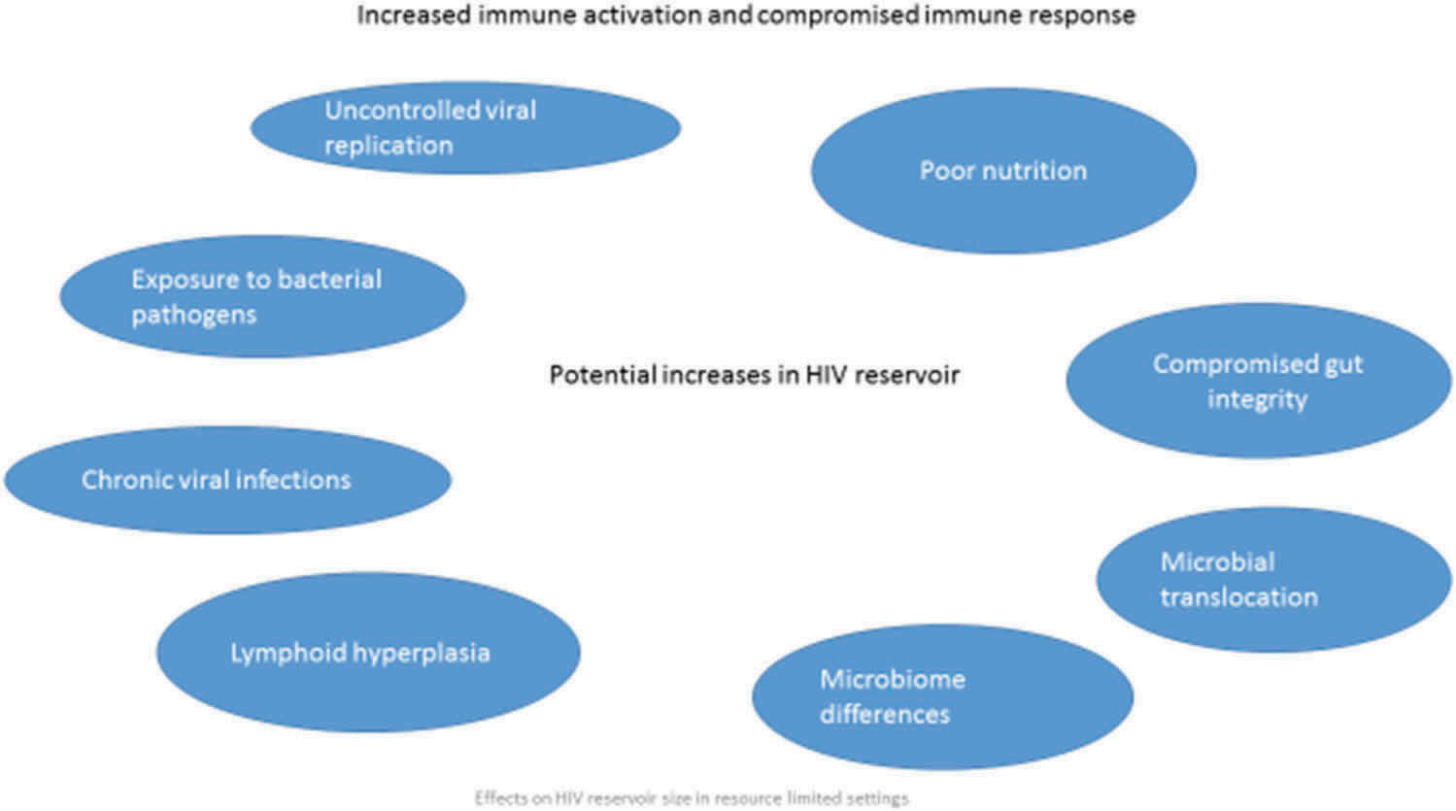
Effects on HIV Reservoir Size in Resource Limited Settings

The cure of the “Berlin patient” combined with prolonged remission in a relative large proportion of early treated individuals in the Visconti cohort and SPARTAC trial has led to enthusiasm for clinical research that aims to achieve HIV remission without continued need for ART. Total eradication of HIV remains a long term but challenging goal. Clinical research in HIV remission and cure research has largely occurred in resource-rich settings where the epidemic is driven by men who have sex with men. In many LMIC, women and children are especially affected by the epidemic. Worldwide, women comprise more than half of all adults living with HIV [3] and there are an estimated 1.8 million children living with HIV [4]. There are important differences in viral strains, HIV-infected populations and the clinical context between resource-rich settings and LMIC. These differences are poorly understood and may be an important barrier to successfully achieving sustained remission or eradication. The size of the pool of latently infected cells, the immune response and viral factors associated with different HIV subtypes may all be important. Indeed, a recent modelling exercise suggested that a cure might be cost-effective in LMIC [5]. If cure research is to reach all HIV-infected people, these differences need to be evaluated and assessed well ahead of any potential interventions. Sex differences that may be important have already been reviewed in this journal [6]. This article focuses on biological factors that may be important in eradication research that are different in different geographic environments.

A critical barrier to achieving sustained remission of HIV is the presence of long-lived latently infected memory CD4+ T cells that contain replication competent HIV DNA, the “reservoir”. A first step towards remission is reducing the reservoir. Factors affecting the reservoir size have not entirely been elucidated, but in LMIC, late presentation to care, the presence of chronic infections causing immune activation and chronic inflammation, increased gut translocation of specific bacterial products and poor nutrition which attenuates the response to infections may be important determinants of the size of the HIV reservoir and may be different in resource-rich and resource-limited environments.

## Methods

We used a scoping review to examine the literature using Arksey and O’Malley’s framework [7]. Scoping studies summarize key evidence on a topic but do not go through the process of a formal systematic review. We searched the following databases: MEDLINE (OVID interface, 1946 onwards), EMBASE (OVID interface, 1947 onwards) and the Cochrane Library. We reviewed this literature to explore biological differences between resource rich and LMIC, focusing on potential differences in the HIV reservoir. No ethics approval was required because this was not human subjects research.

## Immune activation and inflammation

Pre-ART immune activation and inflammation pose an important barrier to reservoir eradication, in several ways: First, inflammation may increase the size and persistence of reservoirs. Patients with higher levels of inflammation and activation before ART initiation have long-lasting higher levels of persisting HIV-infected cells and HIV cell associated RNA [8]. Moreover, chronic inflammation, associated with T-cell activation and proliferation, may result in an increased number of cells harbouring replication competent HIV [9–11]. Immune stimulation also causes lymphoid hyperplasia locally, and the lymphoid follicle has now been shown to be critical to HIV persistence [12]. Integrated HIV DNA, localized in T follicular helper cells within the B-cell follicle, are inaccessible to immune killing at least in part because there are few cytotoxic T lymphocytes (CTLs) with a CD8 phenotype in this compartment. Second, inflammation may limit killing of infected cells, which is an important component of the “kick and kill strategy” for reservoir reduction. T-cell exhaustion, the progressive and irreversible loss of T-cell function, is characterized by decreased effector function, a change in the ability to respond to different antigens and sustained expression of inhibitory receptors such as the checkpoint molecules (for example PD1 and LAG 3) [13–17]. Chronic immune activation is associated with bystander killing of CD4 cells and exhaustion of HIV-1-specific CD4 and CTLs [16,18–20]. Importantly, expression of these markers of immune exhaustion is associated with higher levels of integrated DNA and a more rapid virological rebound in a cohort interrupting therapy suggesting that this process is also associated with an expanded HIV reservoir size [21]. Exhausted CD8 cells are unable to kill HIV-infected cells even when latency is reversed and infected cells express CTL epitopes [22]. While cytokine secretion improves and expression of PD-1 decreases in the presence of durable, suppressive ART [23], functional capacity is not fully restored and multiparametric T-cell pathology persists, despite early initiation of ART [24].

## Uncontrolled HIV

Uncontrolled viral replication is probably the most important factor driving increased immune activation and inflammation in LMIC. Despite changes in guidelines which now recommend therapy for all HIV-infected individuals, full access to HIV treatment is limited. In South Africa, for example, of the estimated 7 million HIV-infected people, only approximately 3 million were on therapy in 2015 [25]. Even when guidelines recommend initiation of therapy, late presentation to care is common. A recent meta-analysis documented profound immunosuppression at ART initiation in many sub-Saharan African countries [26,27]. In addition, although viral load monitoring for clinical care is now recommended, it is not universally available and even when it is available, it is often not acted upon [28]. Uncontrolled HIV replication is associated with a heightened state of inflammation due to both HIV itself and the presence of other infections which are poorly controlled in the setting of immune suppression.

ART initiation at low CD4 counts is often indicative of a prolonged period of uncontrolled HIV and is associated with a greater degree of immune activation [29]. Immune reconstitution is incomplete even after ART has rendered the viral load undetectable, shown in both resource rich and resource poor environments, though there are no direct comparisons [30,31]. Importantly, very early therapy may protect mucosa-associated immune tissue, limit reservoir seeding, prevent CTL escape and retain functional CD4 and CD8 HIV-1 immune responses [32]. Recent work from Thailand has attempted to identify the specific immune lesions in the setting of acute infection and has found that acute HIV infection is associated with increased levels of the biomarkers of intestinal damage, inflammation, coagulation and fibrosis [31]. With suppressive ART initiated during acute HIV infection, the procoagulant state normalizes but gut damage persists and monocyte activation, systemic inflammation and fibrosis remain increased, albeit lower than in individuals starting ART in chronic HIV infection. Importantly, increases in CD4 T-cell populations correlate with decreases in specific markers of immune activation [33]. The notion that immune damage persists after the initial insult was confirmed in a study of biomarkers in the United States [34], and recent data from the AIDS clinical trials group suggest that HIV levels correlate with inflammation and activation before starting therapy but not during long-term suppressive therapy, suggesting that HIV reservoir size is related to factors present before ART initiation [8]. CD8 activation has an important impact on CD4 T-cell recovery [35] and a low CD4/CD8 ratio correlates with T-cell activation; those who start treatment with a low ratio are less likely to normalize over time [36]. The absence of immune exhaustion and premature immune senescence may increase the ability to respond to future therapeutic interventions designed to induce HIV remission [32].

## Viral subtype

Viral subtype may be important in HIV persistence. Globally, subtype C represents about half of all infections and is by far the most common subtype found in LMIC. Subtype B accounts for only approximately 12% of all infections worldwide [37] but the majority of remission-related research is done in patients infected with subtype B virus and there may be significant differences. The HIV tat protein is critical to transcriptional activity and may play an important role in establishing and maintaining latency [38]. Pronounced differences in clade B and C tat protein have been identified [39]; the significance of this to cure strategies has not yet been determined. Investigations into the differences in neurological complications of HIV have revealed that clade C virus is associated with a very different inflammatory environment in the central nervous system and is associated with the expression of different cytokines such as IL-33, leading to different rates of apoptosis of cells infected with clade C virus [40]. Ongoing studies are evaluating the cytokine profiles in patients infected with different subtypes and the differences may be profound [41]. HIV LTR promotors, which are different in different clades, affect transcription of cellular genes. Induction of HIV-1 subtype C viral production may be more responsive to a cellular inflammatory milieu, in particular TNFα production due to an additional NF-κB element in its long terminal repeat (LTR) promoter region [42]. When studying various subtypes and inter-subtype recombinants in Tanzania, subtype C LTR was also associated with a sixfold increased risk of HIV mother to child transmission [43], CD4 cells with HIV DNA integrated near growth genes may proliferate more than other HIV-infected or uninfected cells [44,45]. Studies of cells with identical viral integration sites, indicative of clonal expansion, are currently limited to resource-rich settings and significant differences may exist in LMIC; this is an important knowledge gap that requires research. Importantly, after years on treatment, the integration site repertoire may be affected by the pervasive CD4 cell milieu which may differ in patients with high levels of chronic immune stimulation. It is unknown whether there is link between viral integration and a particular cytokine milieu that affects CD4 proliferation, stimulation and differentiation [46].

## Chronic infections

Many bacterial, viral, and parasitic infections are diseases of poverty and are more common in LMIC. The immune response to these infections may influence the HIV reservoir size when pathogen-specific HIV-infected cells expand in response to antigen stimulation. Chronic antigenic exposure stimulates a broad adaptive and innate immune response. Bacterial infections stimulate reactive lymphoid hyperplasia and growth of local lymph nodes and viral pathogens cause a non-specific T-cell activation that may contribute to the proliferation of T cells containing replication competent HIV. Mounting evidence suggests that macrophages may be infected with HIV and serve as a critical HIV reservoir, most importantly in the central nervous system [47]. The macrophage has diverse roles in host immunity and may be important in the immunopathogenesis of chronic bacterial infections such as tuberculosis (TB). Specific examples of pathogens that are more common in LMIC and affect the populations most impacted by the epidemic may be illustrative of mechanisms that are important in establishing and maintaining the HIV reservoir.

## Tuberculosis (TB)

TB is the most clinically important coinfection for PLHIV in LMIC and is the leading cause of death among PLHIV worldwide [48]. The immune response to TB is characterized by a sustained inflammatory response that persists in many individuals even after therapy for TB has been completed [49]. T-cell activation, as measured by elevated CD38 and HLA-DR expression on CD4+ and CD8+ T-lymphocytes, is described in both latent TB infection and in active TB disease [50]. These lymphocytes can harbour replication competent HIV DNA but it is unknown whether the expansion of these cells in the setting of TB is associated with an increased reservoir size. TB overcomes the intracellular killing mechanisms of the host macrophage’s defences and there may be a synergistic relationship between HIV and TB in which the autophagic pathways are disrupted leading to increased longevity of HIV-infected cells and increased replication of TB in macrophages [51].

## Sexually transmitted bacterial infections

LMIC often have a higher burden of sexually transmitted infections (STIs) and it is plausible that STI coinfection may increase the size of the active HIV reservoir. WHO databases show that antenatal syphilis is more common in LMIC [52]. A systematic review found that 91% of incident infections of chlamydia, gonorrhoea and trichomonas were from individuals in LMIC [53], a finding confirmed by the Global Burden of Disease 2013 Study [54]. Similarly, trichomonas infection is disproportionately found in LMIC [53]. Coinfection with STIs is associated with HIV shedding even when HIV is adequately controlled [55]. One study identified approximately a 2.5-fold risk of HIV shedding in the setting of chlamydia and gonorrhoea in women and a specific cytokine profile in the female genital tract associated with HIV shedding that is also associated with recruitment of CD4 T cells in women seroconverting to HIV [56]. Several studies have found greater HIV shedding in individuals with HIV–trichomonas coinfection [57,58]. Among women with HIV–trichomonas coinfection, trichomonas treatment reduced vaginal HIV shedding at three months post-treatment [58]. Another study from Kenya found a fourfold decrease in vaginal HIV shedding after trichomonas treatment [59]. Both studies included coinfected women who had undetectable HIV viral load in plasma but detectable viral shedding in the genital tract. Although we do not know the implications of these trends for latency, increased shedding may be reflective of an active mucosal reservoir which could pose challenges for immune control and reservoir reduction. Recent work in HIV-uninfected women documented recruitment of potential reservoir cells into the genital tract in women with concomitant STIs, lending credence to the notion that local inflammation may be associated with an expansion of cells that could contain replication competent HIV [60,61]. This is an area that requires further study.

## Other encapsulated bacteria

*Streptococcus pneumoniae* (pneumococcus) and *Haemophilus influenzae* are important pathogens and have a huge disease burden in both HIV-infected and uninfected children. Nasopharyngeal carriage of pneumococcus has been studied in the context of studies of the conjugated pneumococcal vaccine. The prevalence of pneumococcal carriage was as high as 93% in young children [62] in LMIC. In contrast, the highest rates described in resource-rich countries were 50.8% [63]. Higher rates of CD4 cells with memory and effector phenotypes have been described in adenoidal tissue of children colonized with pneumococcus [64]. Rates of *H. influenzae* carriage in HIV-infected children in India were double that of uninfected children [65]. As described above, lymphoid tissue plays a major role in HIV persistence as lymph node follicles are a major HIV reservoir site [14]. The relationship between oropharyngeal carriage of bacterial pathogens, including pneumococcus, and the size of the local HIV reservoir in oropharyngeal mucosa is entirely unknown but may be significant. Given the population structure of the HIV epidemic in LMIC is important to study.

## Viral infections

Chronic viral infections are associated with immune activation and inflammation and are postulated to play an important role in HIV persistence. Modelling research suggests that Africa has a higher burden of both herpes simplex type 2 [66] and herpes simplex type 1 [67] compared to other regions of the world. Other herpesviruses, such as cytomegalovirus (CMV), Epstein Barr virus (EBV) and Kaposi’s sarcoma-associated herpesvirus (KSHV), are endemic in many developing countries [68,69]. CMV seroprevalence approaches 100% in adults in sub-Saharan Africa [70] and EBV and KSHV infections are often acquired very early in life. The role of herpes viruses has been studied in the context of HIV persistence. Asymptomatic shedding of both EBV and CMV viruses is more common in individuals infected with HIV, and recent work has associated more frequent shedding of these viruses in virally suppressed HIV-infected individuals with a larger HIV reservoir [71]. The greatest burden of hepatitis B infection and hepatitis C infection is in LMIC [72–74]. The population attributable fraction for human papillomavirus is 6.9% in less developed regions of the world and 2.1% in more developed regions [75]. The relationship between the HIV reservoir and these viruses is unknown but recent work has shown an association with both inflammatory markers and expansion of T cells with activation and exhaustion markers in women who are dually infected with HIV and HPV [76,77].

## Helminths and other parasites

There is significant geographic overlap between the communities most affected by HIV and helminthic infections. The immunopathology of helminthic infection is complex; they stimulate immune responses via tissue damage and egg production during acute infection but have evolved mechanisms of immune evasion, often using regulatory immune mechanisms. A common feature of all helminthic infections is Th1/Th17-mediated inflammation and Th2-dependent pathology [78,79]. Th2 cells orchestrate the protective immunity against worm infections but, at the same time, the T-regulatory cells induced by helminths appear to attenuate Th2 responses that mediate allergy and autoimmune diseases [80]. Increased levels of immunosuppressive cytokines such as IL-10 and TGF-β1 have been demonstrated [81]. Helminthic infections are associated with the expression of markers of T cell exhaustion [82] and it is plausible that helminthic infestations may contribute to an expansion of the pool of latently infected cells.

## Microbial translocation

One of the key drivers of immune activation in HIV infection is considered to be translocation of intestinal organisms because of early disruption of the intestinal epithelial barrier, due to preferential infection and depletion of CCR5-expressing CD4+ T lymphocytes in the gut associated lymphoid tissue. Portal circulation of these gut organisms may contribute to immune activation [83–87]. Binding of microbial-associated molecular patterns, such as peptidoglycan, lipopolysaccharide, flagellin and CpG DNA, to pattern recognition receptors, activates a signalling cascade in innate immune cells, resulting in the production of pro-inflammatory cytokines, such as interleukin (IL)-1β, IL-6, tumour necrosis factor and type 1 interferons [88,89].

Very early initiation of ART (during stage FI/II) has been shown to maintain the mucosal barrier and fully reverse the initial mucosal and systemic immune activation, most likely through preservation of mucosal CD4+ Th17 cells [90].

The role of intestinal microbiota in aggravating or ameliorating microbial translocation is increasingly recognized [91–93]. HIV has been associated with “bidirectional unbalances [sic]” in metabolic activity of the intestine, potentially affecting the integrity and function of the mucosal barrier as well as local and systemic inflammation [94]. More severe immunosuppression is associated with low diversity microbiota and increased immune activation [95,96] that is present even in people on ART. In LMIC, poor sanitation and hygiene, persistent diarrhoea, inadequate diet and severe acute malnutrition have been described [97] and are associated with inflammation of the small intestine, reduced absorptive capacity, increased intestinal permeability and subsequent increased microbial translocation. Very few studies have examined whether microbial translocation differs between persons living in resource-limited and resource-rich settings. In one study, products of gut translocation were found to be higher in HIV-infected patients from Vietnam and Ethiopia compared to Swedish patients. The researchers proposed in addition to the factors described above that HIV subtype may play a role [98].

## Nutrition

The contribution of nutrition to immunological health cannot be overstated [99]. Inadequate dietary intake leads to a loss of epithelial integrity in the skin, histologically characterized by atrophy and a cutaneous inflammatory response. Malnutrition-associated enteropathy compromises the intestinal barrier function and is accompanied by an inflammatory mucosal infiltrate [100,101]. A Th2 response has been noted and a state of heightened inflammation has been postulated, although a greater proportion of activated T cells has not been found. Intestinal infections, common in LMIC, contribute to micronutrient malabsorption. Vitamins and other micronutrients have been implicated in disease progression and may be important in the microenvironment in which cellular signalling occurs [102,103]. The specific defects that may affect HIV persistence are entirely unknown.

## Laboratory evaluation of the reservoir

There is no universally agreed method for evaluating the size of the latent HIV reservoir [104–106] and different methods either under- or overestimate the pool of cells that contain replication competent DNA. Although often regarded as the gold standard for HIV reservoir assays, as it detects replication competence, the quantitative viral outgrowth assay (QVOA) has significant limitations. It is expensive, cumbersome and requires a large volume of blood and biosafety level 3 procedures. Cell viability is affected by variable processing and storage conditions, although these have improved with standardized procedures and quality management [107]. QVOA may underestimate the number of intact proviruses by 25–27-fold. Only a small proportion of intact proviruses are inducible after one round of stimulation, with additional proviruses being induced after a second round of stimulation [108,109]. Samples that are QVOA negative have been shown to bear infectious virus when using humanized mice for viral recovery [110] and patients who tested negative on QVOA had viral rebound after therapy cessation [111]. Importantly, assay sensitivity is dependent on drawing large volumes of blood to isolate a sufficient number of CD4 cells. Alternative, more practicable assays of inducible provirus are the total virus recovery assay [112] and Tat/rev Induced Limiting Dilution Assay [104]. Although these assays could be useful in monitoring patients receiving curative interventions, they require virus culture facilities and have the limitation that inducible virus does not prove replication competence. Molecular assays of HIV nucleic acid do not require culture facilities and may be more feasible to perform in LMIC: assays of total or integrated HIV DNA can be performed on smaller sample volumes but may severely overestimate the infectious or true reservoir [108]. Low copy viremia detected by a single copy HIV-1 plasma RNA assay correlates well with QVOA but is undetectable in a large proportion of patients [113]. The evaluation of tissue, including lymph nodes and other pathological specimens, requires substantial clinical and laboratory infrastructure which are not available in many LMIC facilities [114,115]. In addition to the above, PCR-based assays require validation across various subtypes and uniform reporting against a reference standard such as the number of cells assayed is required.

## Community response

Community acceptance is critical to a successful cure strategy [116]. Stakeholder engagement among a diverse group of individuals from different local contexts was identified as a priority by the International AIDS Society [117]; yet, communities in regions most affected by the epidemic do not have input into cure studies. Some of the concepts are complex, and messaging needs to be carefully tailored to the specific audience [118]; however, attitudes that suggest that the principles cannot be understood are divisive, presumptuous and unhelpful. HIV remission research will need to be integrated within existing HIV prevention and treatment strategies and prioritized within the context of the overall goal of ending the epidemic. It is likely that focus on immediate prevention and treatment goals is deemed more attainable and more practical than cure goals, but as the field moves forward, access to curative strategies may be delayed because of the need for local safety and efficacy data. Importantly, lack of meaningful participation through mechanisms such as advisory boards could ultimately affect the acceptability of remission strategies [119]. Preparatory research must be implemented in LMIC or the majority of PLHIV globally will not have access to this important strategy.

## Conclusion

The inclusion of patients from high burden LMIC is essential for cure research. HIV subtype diversity, differences in inflammation and concurrent infections, may have an important impact on the size and cell and tissue distribution of the reservoir. The inclusion of patients from high burden LMIC is essential for cure research. Curative strategies that rely on immune function may be sensitive to differences in immune exhaustion, and the determinants of immune exhaustion in diverse settings need to be studied. We outline specific areas for research (Table 1), but it is clear that including patients from LMIC early in the process of clinical research might then accelerate the gradual process of scaling up an effective HIV cure in the future. In the SPARTAC study, a high proportion (22.7%) of early treated African patients achieved post-therapy control [120] demonstrating that treatment goals are feasible and acceptable. Early treatment would reduce the burden of opportunistic infections associated with delayed initiation and prevent onward transmission from early acute hyper-infectious individuals. Such an approach towards remission should be prioritized in LMIC.

**Table 1.** Knowledge gaps regarding HIV remission research in low- and middle-income countries

Role of HIV subtype in viral integration, transcriptional activation and CD4+ T cell growth
Effect of a particular cytokine milieu that affects CD4 proliferation, stimulation and differentiation on viral integration
Whether TB-induced expansion of T cells increases HIV reservoir size
Association between chronic viral infections, such as hepatitis B, hepatitis C and HPV, and the HIV reservoir
The relationship between oropharyngeal carriage of bacterial pathogens and the size of the local HIV reservoir in oropharyngeal mucosa
Whether viral shedding in the presence of coinfections, such as trichomonas, impacts of measures to achieve latency
Impact of poor nutrition on immune function, activation and the size of the HIV reservoir
Biomarker of HIV reservoir size available in LMIC

## References

[1] UNAIDS AIDS by the Numbers: UNAIDS; 2016 Available from: http://www.unaids.org/en/resources/documents/2016/AIDS-by-the-numbers

[2] World Health Organization. Consolidated Guidelines on the Use of Antiretroviral Drugs for Treating and Preventing HIV Infection: recommendations for a Public Health Approach. Geneva: WHO; 2016 Available from: http://www.who.int/hiv/pub/arv/arv-2016/en/. Accessed 24/5/2017. 27466667

[3] Women U Facts and figures: HIV and AIDS 2016 [updated 2016 6 Available from: http://www.unwomen.org/en/what-we-do/hiv-and-aids/facts-and-figures#notes

[4] UNICEF UNICEF data: monitoring the situation of children and women. 2016 [updated 2016 12]. Available from: https://data.unicef.org/topic/hivaids/global-regional-trends/

[5] Phillips A, Cambiano V, Revill P, Nakagawa F, Lundgren J, Bansi-Matharu L, et al Identifying key drivers of the impact of an HIV cure intervention in sub-Saharan Africa. J Infect Dis. 2016;214:73–9. 27034345 10.1093/infdis/jiw120 PMC4907418

[6] Gianella S, Tsibris A, Barr L, Godfrey C. Barriers to a cure for HIV in women. J Int AIDS Soc. 2016;19(1):20706. 26900031 10.7448/IAS.19.1.20706 PMC4761692

[7] Arksey H, O’Malley L Scoping studies: towards a methodological framework. Int J Soc Res Methodol. 2005;8(1):19–32.

[8] Gandhi RT, McMahon DK, Bosch RJ, Lalama C, Macatangay B, Cyktor J, et al Levels of HIV-1 persistence on antiretroviral therapy are not associated with markers of inflammation or activation. PLoS Pathog. 2017;13:e1006285. 28426825 10.1371/journal.ppat.1006285 PMC5398724

[9] Hatano H, Jain V, Hunt PW, Lee TH, Sinclair E, Do TD, et al Cell-based measures of viral persistence are associated with immune activation and programmed cell death protein 1 (PD-1)-expressing CD4+ T cells. J Infect Dis. 2013;208(1):50–56. 23089590 10.1093/infdis/jis630 PMC3666131

[10] Cockerham LR, Siliciano JD, Sinclair E, O’Doherty U, Palmer S, Yukl SA, et al CD4+ and CD8+ T cell activation are associated with HIV DNA in resting CD4+ T cells. Plos One. 2014;9(10):e110731. 25340755 10.1371/journal.pone.0110731 PMC4207702

[11] Massanella M, Fromentin R, Chomont N Residual inflammation and viral reservoirs: alliance against an HIV cure. Curr Opin HIV AIDS. 2016;11(2):234–41. 26575148 10.1097/COH.0000000000000230 PMC4743501

[12] Miles B, Connick E TFH in HIV latency and as sources of replication-competent virus. Trends Microbiol. 2016;24(5):338–44. 26947191 10.1016/j.tim.2016.02.006 PMC4841704

[13] Wherry EJ T cell exhaustion. Nat Immunol. 2011;12(6):492–99. 21739672 10.1038/ni.2035

[14] Banga R, Procopio FA, Noto A, Pollakis G, Cavassini M, Ohmiti K, et al PD-1(+) and follicular helper T cells are responsible for persistent HIV-1 transcription in treated aviremic individuals. Nat Med. 2016;22(7):754–61. 27239760 10.1038/nm.4113

[15] Connick E, Mattila T, Folkvord JM, Schlichtemeier R, Meditz AL, Ray MG, et al CTL fail to accumulate at sites of HIV-1 replication in lymphoid tissue. J Immunol (Baltimore, Md 1950). 2007;178(11):6975–83. 10.4049/jimmunol.178.11.6975 17513747

[16] Kahan SM, Wherry EJ, Zajac AJ T cell exhaustion during persistent viral infections. Virology. 2015;479-480:180–93. 25620767 10.1016/j.virol.2014.12.033 PMC4424083

[17] Kohler SL, Pham MN, Folkvord JM, Arends T, Miller SM, Miles B, et al Germinal center T follicular helper cells are highly permissive to HIV-1 and alter their phenotype during virus replication. J Immunol (Baltimore, Md 1950). 2016;196(6):2711–22. 10.4049/jimmunol.1502174 PMC4779697 26873986

[18] Wherry EJ, Kurachi M Molecular and cellular insights into T cell exhaustion. Nat Rev Immunol. 2015;15(8):486–99. 26205583 10.1038/nri3862 PMC4889009

[19] Shive CL, Clagett B, McCausland MR, Mudd JC, Funderburg NT, Freeman ML, et al Inflammation perturbs the IL-7 axis, promoting senescence and exhaustion that broadly characterize immune failure in treated HIV infection. J Acquir Immune Defic Syndr. 2016;71(5):483–92. 26627102 10.1097/QAI.0000000000000913 PMC4788576

[20] Bui JK, Mellors JW Reversal of T-cell exhaustion as a strategy to improve immune control of HIV-1. AIDS. 2015;29(15):1911–15. 26355569 10.1097/QAD.0000000000000788

[21] Hurst J, Hoffmann M, Pace M, Williams JP, Thornhill J, Hamlyn E, et al Immunological biomarkers predict HIV-1 viral rebound after treatment interruption. Nat Commun. 2015;6:8495. 26449164 10.1038/ncomms9495 PMC4633715

[22] Shan L, Siliciano RF From reactivation of latent HIV-1 to elimination of the latent reservoir: the presence of multiple barriers to viral eradication. Bioessays. 2013;35(6):544–52. 23613347 10.1002/bies.201200170 PMC4386637

[23] Rehr M, Cahenzli J, Haas A, Price DA, Gostick E, Huber M, et al Emergence of polyfunctional CD8+ T cells after prolonged suppression of human immunodeficiency virus replication by antiretroviral therapy. J Virol. 2008;82(7):3391–404. 18199637 10.1128/JVI.02383-07 PMC2268491

[24] Tauriainen J, Scharf L, Frederiksen J, Naji A, Ljunggren HG, Sonnerborg A, et al Perturbed CD8+ T cell TIGIT/CD226/PVR axis despite early initiation of antiretroviral treatment in HIV infected individuals. Sci Rep. 2017;7:40354. 28084312 10.1038/srep40354 PMC5233961

[25] PEPFAR Pepfar dashboards. 2015 Available from: https://data.pepfar.net/global

[26] Siedner MJ, Ng CK, Bassett IV, Katz IT, Bangsberg DR, Tsai AC Trends in CD4 count at presentation to care and treatment initiation in Sub-Saharan Africa, 2002–2013: a meta-analysis. Clin Infect Dis: Off Publ Infect Dis Soc America. 2015;60(7):1120–27. 10.1093/cid/ciu1137 PMC4366582 25516189

[27] Ford N, Mills EJ, Egger M Editorial commentary: immunodeficiency at start of antiretroviral therapy: the persistent problem of late presentation to care. Clin Infect Dis: Off Publ Infect Dis Soc America. 2015;60(7):1128–30. 10.1093/cid/ciu1138 PMC4357289 25516184

[28] Petersen ML, Tran L, Geng EH, Reynolds SJ, Kambugu A, Wood R, et al Delayed switch of antiretroviral therapy after virologic failure associated with elevated mortality among HIV-infected adults in Africa. AIDS (London, England). 2014;28(14):2097–107. 10.1097/QAD.0000000000000349 PMC4317283 24977440

[29] Skogmar S, Schön T, Balcha TT, Sturegård E, Jansson M, Björkman P, et al plasma levels of neopterin and C-reactive protein (CRP) in tuberculosis (TB) with and without HIV coinfection in relation to CD4 cell count. Plos One. 2015;10(12):e0144292. 26630153 10.1371/journal.pone.0144292 PMC4668010

[30] Guo F-P, Li Y-J, Qiu Z-F, Lv W, Han Y, Xie J, et al Baseline naive CD4+ T-cell level predicting immune reconstitution in treated HIV-infected late presenters. Chin Med J. 2016;129(22):2683–90. 27824000 10.4103/0366-6999.193460 PMC5126159

[31] Robbins GK, Spritzler JG, Chan ES, Asmuth DM, Gandhi RT, Rodriguez BA, et al Incomplete reconstitution of T cell subsets on combination antiretroviral therapy in the AIDS Clinical Trials Group protocol 384. Clin Infect Dis. 2009;48(3):350–61. 19123865 10.1086/595888 PMC2676920

[32] Krebs SJ, Ananworanich J Immune activation during acute HIV infection and the impact of early antiretroviral therapy. Curr Opin HIV AIDS. 2016;11(2):163–72. 26599167 10.1097/COH.0000000000000228

[33] Sereti I, Krebs SJ, Phanuphak N, Fletcher JL, Slike B, Pinyakorn S, et al Persistent, albeit reduced, chronic inflammation in persons starting antiretroviral therapy in acute HIV infection. Clin Infect Dis. 2017;64(2):124–31. 27737952 10.1093/cid/ciw683 PMC5215214

[34] Macatangay BJ, Yang M, Sun X, Morton J, Gruttola V, Little S, et al Changes in levels of inflammation following antiretroviral treatment during early HIV infection in ACTG A5217. J Acquir Immune Defic Syndr. 2017;75:137–41. 28198712 10.1097/QAI.0000000000001320 PMC5393630

[35] Hunt PW, Cao HL, Muzoora C, Ssewanyana I, Bennett J, Emenyonu N, et al Impact of CD8+ T cell activation on CD4+ T cell recovery and mortality in HIV-infected Ugandans initiating antiretroviral therapy. AIDS (London, England). 2011;25(17):2123–31. 10.1097/QAD.0b013e32834c4ac1 PMC3480326 21881481

[36] Bellissimo F, Pinzone MR, Celesia BM, Cacopardo B, Nunnari G Baseline CD4/CD8 T-cell ratio predicts prompt immune restoration upon cART initiation. Curr HIV Res. 2016;14:491–96. 27074946 10.2174/1570162x14666160414111554

[37] Hemelaar J The origin and diversity of the HIV-1 pandemic. Trends Mol Med. 2012;18(3):182–92. 22240486 10.1016/j.molmed.2011.12.001

[38] Razooky BS, Pai A, Aull K, Rouzine IM, Weinberger LS A hardwired HIV latency program. Cell. 2015;160(5):990–1001. 25723172 10.1016/j.cell.2015.02.009 PMC4395878

[39] Zhao X, Qian L, Zhou D, Qi D, Liu C, Kong X Stability of HIV-1 subtype B and C Tat is associated with variation in the carboxyl-terminal region. Virol Sin. 2016;31(3):199–206. 27007880 10.1007/s12250-016-3681-0 PMC8193441

[40] Yndart A, Kaushik A, Agudelo M, Raymond A, Atluri VS, Saxena SK, et al Investigation of neuropathogenesis in HIV-1 clade B and C infection associated with IL-33 and ST2 regulation. ACS Chem Neurosci. 2015;6(9):1600–12. 26110635 10.1021/acschemneuro.5b00156

[41] Tm R HIV-1-induced immune activation as a predictor of treatment outcome in patients receiving highly-active antiretroviral therapy.[Submitted in fulfilment of the requirements of the degree of Doctor of Philosophy]. University of Pretoria; 2016.

[42] Montano MA, Nixon CP, Ndung’u T, Bussmann H, Novitsky VA, Dickman D, et al Elevated tumor necrosis factor-alpha activation of human immunodeficiency virus type 1 subtype C in Southern Africa is associated with an NF-kappaB enhancer gain-of-function. J Infect Dis. 2000;181(1):76–81. 10608753 10.1086/315185

[43] Blackard JT, Renjifo B, Fawzi W, Hertzmark E, Msamanga G, Mwakagile D, et al HIV-1 LTR subtype and perinatal transmission. Virology. 2001;287(2):261–65. 11531404 10.1006/viro.2001.1059

[44] Wagner TA, McLaughlin S, Garg K, Cheung CY, Larsen BB, Styrchak S, et al HIV latency. Proliferation of cells with HIV integrated into cancer genes contributes to persistent infection. Science. 2014;345(6196):570–73. 25011556 10.1126/science.1256304 PMC4230336

[45] Maldarelli F, Wu X, Su L, Simonetti FR, Shao W, Hill S, et al HIV latency. Specific HIV integration sites are linked to clonal expansion and persistence of infected cells. Science. 2014;345(6193):179–83. 24968937 10.1126/science.1254194 PMC4262401

[46] Zhang H, Jiao Y, Li H, Zhu W, Li W, Huang X, et al Longitudinal changes in total, 2-LTR circular, and integrated HIV-1 DNA during the first year of HIV-1 infection in CD4Low and CD4High patient groups with HIV-1 subtype AE. Viral Immunol. 2014;27(9):478–82. 25188292 10.1089/vim.2014.0064

[47] Hellmuth J, Valcour V, Spudich S CNS reservoirs for HIV: implications for eradication. J Virus Eradication. 2015;1(2):67–71. 10.1016/S2055-6640(20)30489-1 PMC4586130 26430703

[48] Organization WH 2016 [cited 2016 98]. Available from: http://www.who.int/mediacentre/factsheets/fs104/en/

[49] Young JM, Adetifa IM, Ota MO, Sutherland JS Expanded polyfunctional T cell response to mycobacterial antigens in TB disease and contraction post-treatment. Plos One. 2010;5(6):e11237. 20574540 10.1371/journal.pone.0011237 PMC2888639

[50] Sullivan ZA, Wong EB, Ndung’u T, Kasprowicz VO, Bishai WR Latent and active tuberculosis infection increase immune activation in individuals co-infected with HIV. EBioMedicine. 2015;2(4):334–40. 26114158 10.1016/j.ebiom.2015.03.005 PMC4476549

[51] Espert L, Beaumelle B, Vergne I Autophagy in Mycobacterium tuberculosis and HIV infections. Front Cell Infect Microbiol. 2015;5:49. 26082897 10.3389/fcimb.2015.00049 PMC4451423

[52] Newman L, Kamb M, Hawkes S, Gomez G, Say L, Seuc A, et al Global estimates of syphilis in pregnancy and associated adverse outcomes: analysis of multinational antenatal surveillance data. Plos Med. 2013;10(2):e1001396. 23468598 10.1371/journal.pmed.1001396 PMC3582608

[53] Newman L, Rowley J, Vander Hoorn S, Wijesooriya NS, Unemo M, Low N, et al Global estimates of the prevalence and incidence of four curable sexually transmitted infections in 2012 based on systematic review and global reporting. Plos One. 2015;10(12):e0143304. 26646541 10.1371/journal.pone.0143304 PMC4672879

[54] Global Burden of Disease Study C Global, regional, and national incidence, prevalence, and years lived with disability for 301 acute and chronic diseases and injuries in 188 countries, 1990-2013: a systematic analysis for the Global Burden of Disease Study 2013. Lancet. 2015;386(9995):743–800. 26063472 10.1016/S0140-6736(15)60692-4 PMC4561509

[55] Mitchell C, Hitti J, Paul K, Agnew K, Cohn SE, Luque AE, et al Cervicovaginal shedding of HIV type 1 is related to genital tract inflammation independent of changes in vaginal microbiota. AIDS Res Hum Retroviruses. 2011;27(1):35–39. 20929397 10.1089/aid.2010.0129 PMC3034096

[56] Mauck C, Chen PL, Morrison CS, Fichorova RN, Kwok C, Chipato T, et al Biomarkers of cervical inflammation and immunity associated with cervical shedding of HIV-1. AIDS Res Hum Retroviruses. 2016;32(5):443–51. 26650885 10.1089/aid.2015.0088 PMC4845652

[57] Tanton C, Weiss HA, Le Goff J, Changalucha J, Rusizoka M, Baisley K, et al Correlates of HIV-1 genital shedding in Tanzanian women. Plos One. 2011;6(3):e17480. 21390251 10.1371/journal.pone.0017480 PMC3046975

[58] Kissinger P, Amedee A, Clark RA, Dumestre J, Theall KP, Myers L, et al Trichomonas vaginalis treatment reduces vaginal HIV-1 shedding. Sex Transm Dis. 2009;36(1):11–16. 19008776 10.1097/OLQ.0b013e318186decf PMC3779369

[59] Wang CC, McClelland RS, Reilly M, Overbaugh J, Emery SR, Mandaliya K, et al The effect of treatment of vaginal infections on shedding of human immunodeficiency virus type 1. J Infect Dis. 2001;183(7):1017–22. 11237825 10.1086/319287

[60] Anahtar MN, Byrne EH, Doherty KE, Bowman BA, Yamamoto HS, Soumillon M, et al Cervicovaginal bacteria are a major modulator of host inflammatory responses in the female genital tract. Immunity. 2015;42(5):965–76. 25992865 10.1016/j.immuni.2015.04.019 PMC4461369

[61] Passmore JA, Jaspan HB, Masson L Genital inflammation, immune activation and risk of sexual HIV acquisition. Curr Opin HIV AIDS. 2016;11(2):156–62. 26628324 10.1097/COH.0000000000000232 PMC6194860

[62] Adegbola RA, DeAntonio R, Hill PC, Roca A, Usuf E, Hoet B, et al Carriage of Streptococcus pneumoniae and other respiratory bacterial pathogens in low and lower-middle income countries: a systematic review and meta-analysis. Plos One. 2014;9(8):e103293. 25084351 10.1371/journal.pone.0103293 PMC4118866

[63] Bogaert D, de Groot R, Hermans PWM Streptococcus pneumoniae colonisation: the key to pneumococcal disease. Lancet Infect Dis. 2004;4(3):144–54. 14998500 10.1016/S1473-3099(04)00938-7

[64] Zhang Q, Leong SC, McNamara PS, Mubarak A, Malley R, Finn A Characterisation of regulatory T cells in nasal associated lymphoid tissue in children: relationships with pneumococcal colonization. Plos Pathog. 2011;7(8):e1002175. 21852948 10.1371/journal.ppat.1002175 PMC3154846

[65] Arya BK, Bhattacharya SD, Sutcliffe C, Niyogi SK, Bhattacharyya S, Hemram S, et al Impact of haemophilus influenzae type b conjugate vaccines (HibCV) on nasopharyngeal carriage in HIV infected children and their parents from West Bengal, India. Pediatr Infect Dis J. 2016;35:e339-e347. 27753766 10.1097/INF.0000000000001266

[66] Looker KJ, Magaret AS, Turner KM, Vickerman P, Gottlieb SL, Newman LM Global estimates of prevalent and incident herpes simplex virus type 2 infections in 2012. Plos One. 2015;10(1):e114989. 25608026 10.1371/journal.pone.0114989 PMC4301914

[67] Looker KJ, Magaret AS, May MT, Turner KM, Vickerman P, Gottlieb SL, et al Global and regional estimates of prevalent and incident herpes simplex virus type 1 infections in 2012. Plos One. 2015;10(10):e0140765. 26510007 10.1371/journal.pone.0140765 PMC4624804

[68] Akbar AN, Fletcher JM Memory T cell homeostasis and senescence during aging. Curr Opin Immunol. 2005;17(5):480–85. 16098721 10.1016/j.coi.2005.07.019

[69] Hunt PW HIV and inflammation: mechanisms and consequences. Curr HIV/AIDS Rep. 2012;9(2):139–47. 22528766 10.1007/s11904-012-0118-8

[70] Adland E, Klenerman P, Goulder P, Matthews PC Ongoing burden of disease and mortality from HIV/CMV coinfection in Africa in the antiretroviral therapy era. Front Microbiol. 2015;6:1016. 26441939 10.3389/fmicb.2015.01016 PMC4585099

[71] Gianella S, Anderson CM, Var SR, Oliveira MF, Lada SM, Vargas MV, et al Replication of human herpesviruses is associated with higher HIV DNA levels during antiretroviral therapy started at early phases of HIV infection. J Virol. 2016;90(8):3944–52. 26842469 10.1128/JVI.02638-15 PMC4810527

[72] Gower E, Estes C, Blach S, Razavi-Shearer K, Razavi H Global epidemiology and genotype distribution of the hepatitis C virus infection. J Hepatol. 2014;61(1 Suppl):S45–57. 25086286 10.1016/j.jhep.2014.07.027

[73] Ott JJ, Stevens GA, Groeger J, Wiersma ST Global epidemiology of hepatitis B virus infection: new estimates of age-specific HBsAg seroprevalence and endemicity. Vaccine. 2012;30(12):2212–19. 22273662 10.1016/j.vaccine.2011.12.116

[74] Mandeville KL, Krabshuis J, Ladep NG, Mulder CJ, Quigley EM, Khan SA Gastroenterology in developing countries: issues and advances. World J Gastroenterol. 2009;15(23):2839–54. 19533805 10.3748/wjg.15.2839 PMC2699001

[75] Forman D, de Martel C, Lacey CJ, Soerjomataram I, Lortet-Tieulent J, Bruni L, et al Global burden of human papillomavirus and related diseases. Vaccine. 2012;30(Suppl 5):F12–23. 23199955 10.1016/j.vaccine.2012.07.055

[76] Buckley N, Huber A, Lo Y, Castle PE, Kemal K, Burk RD, et al Association of high-risk human papillomavirus with genital tract mucosal immune factors in HIV-infected women. Am J Reprod Immunol (New York, NY 1989). 2016;75(2):146–54. 10.1111/aji.12461 PMC4715979 26685115

[77] Papasavvas E, Surrey LF, Glencross DK, Azzoni L, Joseph J, Omar T, et al High-risk oncogenic HPV genotype infection associates with increased immune activation and T cell exhaustion in ART-suppressed HIV-1-infected women. OncoImmunology. 2016;5(5):e1128612. 27467943 10.1080/2162402X.2015.1128612 PMC4910732

[78] George PJ, Kumar NP, Sridhar R, Hanna LE, Nair D, Banurekha VV, et al Coincident helminth infection modulates systemic inflammation and immune activation in active pulmonary tuberculosis. Plos Negl Trop Dis. 2014;8(11):e3289. 25375117 10.1371/journal.pntd.0003289 PMC4222842

[79] Finlay CM, Walsh KP, Mills KHG Induction of regulatory cells by helminth parasites: exploitation for the treatment of inflammatory diseases. Immunol Rev. 2014;259(1):206–30. 24712468 10.1111/imr.12164

[80] Maizels RM, Yazdanbakhsh M T-cell regulation in helminth parasite infections: implications for inflammatory diseases. Chem Immunol Allergy. 2008;94:112–23. 18802342 10.1159/000154944

[81] Borkow G, Bentwich Z Chronic immune activation associated with chronic helminthic and human immunodeficiency virus infections: role of hyporesponsiveness and anergy. Clin Microbiol Rev. 2004;17(4):1012–30. table of contents. 15489359 10.1128/CMR.17.4.1012-1030.2004 PMC523563

[82] Zander RA, Butler NS Dysfunctional adaptive immunity during parasitic infections. Curr Immunol Rev. 2013;9(3):179–89. 24839433 10.2174/1573395509666131126230832 PMC4020283

[83] Mehandru S, Ma P, Tenner-Racz K, Jean-Pierre P, Manuelli V, Lopez P, et al Lack of mucosal immune reconstitution during prolonged treatment of acute and early HIV-1 infection. Plos Med. 2006;3(12):e484. 17147468 10.1371/journal.pmed.0030484 PMC1762085

[84] Hayes TL, Asmuth DM, Critchfield JW, Knight TH, McLaughlin BE, Yotter T, et al Impact of highly active antiretroviral therapy initiation on CD4(+) T-cell repopulation in duodenal and rectal mucosa. AIDS. 2013;27(6):867–77. 23262500 10.1097/QAD.0b013e32835d85b4 PMC4026034

[85] Gordon SN, Cervasi B, Odorizzi P, Silverman R, Aberra F, Ginsberg G, et al Disruption of intestinal CD4+ T cell homeostasis is a key marker of systemic CD4+ T cell activation in HIV-infected individuals. J Immunol. 2010;185(9):5169–79. 20889546 10.4049/jimmunol.1001801 PMC3155848

[86] Brenchley JM, Douek DC Microbial translocation across the GI tract. Annu Rev Immunol. 2012;30:149–73. 22224779 10.1146/annurev-immunol-020711-075001 PMC3513328

[87] Mavigner M, Cazabat M, Dubois M, L’Faqihi FE, Requena M, Pasquier C, et al Altered CD4+ T cell homing to the gut impairs mucosal immune reconstitution in treated HIV-infected individuals. J Clin Invest. 2012;122(1):62–69. 22156200 10.1172/JCI59011 PMC3248296

[88] Hayashi F, Smith KD, Ozinsky A, Hawn TR, Yi EC, Goodlett DR, et al The innate immune response to bacterial flagellin is mediated by Toll-like receptor 5. Nature. 2001;410(6832):1099–103. 11323673 10.1038/35074106

[89] Sandler NG, Douek DC Microbial translocation in HIV infection: causes, consequences and treatment opportunities. Nat Rev Microbiol. 2012;10(9):655–66. 22886237 10.1038/nrmicro2848

[90] Schuetz A, Deleage C, Sereti I, Rerknimitr R, Phanuphak N, Phuang-Ngern Y, et al Initiation of ART during early acute HIV infection preserves mucosal Th17 function and reverses HIV-related immune activation. Plos Pathog. 2014;10(12):e1004543. 25503054 10.1371/journal.ppat.1004543 PMC4263756

[91] Gori A, Tincati C, Rizzardini G, Torti C, Quirino T, Haarman M, et al Early impairment of gut function and gut flora supporting a role for alteration of gastrointestinal mucosa in human immunodeficiency virus pathogenesis. J Clin Microbiol. 2008;46(2):757–58. 18094140 10.1128/JCM.01729-07 PMC2238068

[92] Ellis CL, Ma ZM, Mann SK, Li CS, Wu J, Knight TH, et al Molecular characterization of stool microbiota in HIV-infected subjects by panbacterial and order-level 16S ribosomal DNA (rDNA) quantification and correlations with immune activation. J Acquir Immune Defic Syndr. 2011;57(5):363–70. 21436711 10.1097/QAI.0b013e31821a603c PMC3153564

[93] Vujkovic-Cvijin I, Dunham RM, Iwai S, Maher MC, Albright RG, Broadhurst MJ, et al Dysbiosis of the gut microbiota is associated with HIV disease progression and tryptophan catabolism. Sci Transl Med. 2013;5(193):193ra91. 10.1126/scitranslmed.3006438 PMC4094294 23843452

[94] Guillén YN-JM, Muntsa Rocafort M, Mariona Parera M, Casadellà M, Bravo I, Coll J, et al Functional profiling of the gut microbiome in HIV infection. Seattle (WA): CROI; 2017.

[95] Mutlu EA, Keshavarzian A, Losurdo J, Swanson G, Siewe B, Forsyth C, et al A compositional look at the human gastrointestinal microbiome and immune activation parameters in HIV infected subjects. Plos Pathog. 2014;10(2):e1003829. 24586144 10.1371/journal.ppat.1003829 PMC3930561

[96] Monaco CL, Gootenberg DB, Zhao G, Handley SA, Ghebremichael MS, Lim ES, et al Altered virome and bacterial microbiome in human immunodeficiency virus-associated acquired immunodeficiency syndrome. Cell Host Microbe. 2016;19(3):311–22. 26962942 10.1016/j.chom.2016.02.011 PMC4821831

[97] Prendergast A, Kelly P Enteropathies in the developing world: neglected effects on global health. Am J Trop Med Hyg. 2012;86(5):756–63. 22556071 10.4269/ajtmh.2012.11-0743 PMC3335677

[98] Abdurahman S, Barqasho B, Nowak P, Cuong Do D, Amogne W, Larsson M, et al Pattern of microbial translocation in patients living with HIV-1 from Vietnam, Ethiopia and Sweden. J Int AIDS Soc. 2014;17:18841. 24461466 10.7448/IAS.17.1.18841 PMC3902178

[99] Katona P, Katona-Apte J The interaction between nutrition and infection. Clin Infect Dis. 2008;46(10):1582–88. 18419494 10.1086/587658

[100] Prendergast AJ Malnutrition and vaccination in developing countries. Philosophical Trans Royal Soc B: Biol Sci. 2015;370(1671):20140141. 10.1098/rstb.2014.0141 PMC4527386 25964453

[101] Rytter MJ, Kolte L, Briend A, Friis H, Christensen VB The immune system in children with malnutrition–a systematic review. Plos One. 2014;9(8):e105017. 25153531 10.1371/journal.pone.0105017 PMC4143239

[102] Amare B, Moges B, Mulu A, Yifru S, Kassu A Quadruple burden of HIV/AIDS, tuberculosis, chronic intestinal parasitoses, and multiple micronutrient deficiency in ethiopia: a summary of available findings. Biomed Res Int. 2015;2015:598605. 25767808 10.1155/2015/598605 PMC4342072

[103] Abhimanyu MV, Jeffery TJ, Vitamin BL D status in South Africa and tuberculosis. Lung. 2015;193(6):975–84. 26307505 10.1007/s00408-015-9789-4

[104] Procopio FA, Fromentin R, Kulpa DA, Brehm JH, Bebin AG, Strain MC, et al A novel assay to measure the magnitude of the inducible viral reservoir in HIV-infected individuals. EBioMedicine. 2015;2(8):874–83. 26425694 10.1016/j.ebiom.2015.06.019 PMC4563128

[105] Bruner KM, Hosmane NN, Siliciano RF Towards an HIV-1. cure: measuring the latent reservoir. Trends in microbiology; 2015. 10.1016/j.tim.2015.01.013 PMC4386620 25747663

[106] Hong F, Aga E, Cillo AR, Yates AL, Besson G, Fyne E, et al Novel assays for measurement of total cell-associated HIV-1 DNA and RNA. J Clin Microbiol. 2016;54(4):902–11. 26763968 10.1128/JCM.02904-15 PMC4809955

[107] Sanchez AM, Denny TN, O’Gorman M Introduction to a special issue of the journal of immunological methods: building global resource programs to support HIV/AIDS clinical trial studies. J Immunol Methods. 2014;409:1–5. 24910413 10.1016/j.jim.2014.05.016 PMC4138253

[108] Ho YC, Shan L, Hosmane NN, Wang J, Laskey SB, Rosenbloom DI, et al Replication-competent noninduced proviruses in the latent reservoir increase barrier to HIV-1 cure. Cell. 2013;155(3):540–51. 24243014 10.1016/j.cell.2013.09.020 PMC3896327

[109] Bruner KM, Murray AJ, Pollack RA, Soliman MG, Laskey SB, Capoferri AA, et al Defective proviruses rapidly accumulate during acute HIV-1 infection. Nat Med. 2016;22(9):1043–49. 27500724 10.1038/nm.4156 PMC5014606

[110] Metcalf Pate KA, Pohlmeyer CW, Walker-Sperling VE, Foote JB, Najarro KM, Cryer CG, et al A murine viral outgrowth assay to detect residual HIV type 1 in patients with undetectable viral loads. J Infect Dis. 2015;212(9):1387–96. 25883388 10.1093/infdis/jiv230 PMC4601916

[111] Rainwater-Lovett K, Luzuriaga K, Persaud D Very early combination antiretroviral therapy in infants: prospects for cure. Curr Opin HIV AIDS. 2015;10(1):4–11. 25402708 10.1097/COH.0000000000000127 PMC4351817

[112] Anthony CMS, Buckley T, John Bui JCC, Mellors JW Latent HIV is missed by assaying only resting CD4+ T cells. Paper presented at: Conference on Retroviruses and Opportunistic Infections (CROI), Seattle (WA); 2017.

[113] Crooks AM, Bateson R, Cope AB, Dahl NP, Griggs MK, Kuruc JD, et al Precise quantitation of the latent HIV-1 reservoir: implications for eradication strategies. J Infect Dis. 2015;212(9):1361–65. 25877550 10.1093/infdis/jiv218 PMC4601910

[114] Adesina A, Chumba D, Nelson AM, Orem J, Roberts DJ, Wabinga H, et al Improvement of pathology in sub-Saharan Africa. Lancet Oncol. 2013;14(4):e152–7. 23561746 10.1016/S1470-2045(12)70598-3

[115] Adewole I, Martin DN, Williams MJ, Adebamowo C, Bhatia K, Berling C, et al Building capacity for sustainable research programmes for cancer in Africa. Nat Rev Clin Oncol. 2014;11(5):251–59. 24614139 10.1038/nrclinonc.2014.37 PMC4403794

[116] Tucker JD, Rennie S Ethical Working Group on HIVC. Social and ethical implications of HIV cure research. AIDS. 2014;28:1247–50. 24451161 10.1097/QAD.0000000000000210 PMC4071118

[117] Deeks SG, Lewin SR, Ross AL, Ananworanich J, Benkirane M, Cannon P, et al International AIDS Society global scientific strategy: towards an HIV cure 2016. Nat Med. 2016;22(8):839–50. 27400264 10.1038/nm.4108 PMC5322797

[118] Tucker JD, Volberding PA, Margolis DM, Rennie S, Barré-Sinoussi F Words matter: discussing research towards an HIV cure in research and clinical contexts. J Acquir Immune Defic Syndr. 2014;67(3):e110–1. 25314254 10.1097/QAI.0000000000000305 PMC4199082

[119] Lo Y, Chu C, Ananworanich J, Excler J, Tucker JD Stakeholder engagement in HIV cure research: lessons learned from other HIV interventions and the way forward. AIDS Patient Care & Stds. 2015;29:389–99. 26061668 10.1089/apc.2014.0348 PMC4504439

[120] Gossez M, Ramjee G, Hurst J, Kaleebu P, Rees H, Porter K, et al Virological remission after ART interruption in African HIV-1 seroconverters. In: CROI 2016 Conference on Retroviruses and Opportunistic Infections (CROI); 2016 2 22–25; Boston (MA).

